# Strengthening primary eye care in South Africa: An assessment of services and prospective evaluation of a health systems support package

**DOI:** 10.1371/journal.pone.0197432

**Published:** 2018-05-14

**Authors:** Rivka R. Lilian, Jean Railton, Erik Schaftenaar, Moyahabo Mabitsi, Cornelis J. Grobbelaar, N. Sellina Khosa, Babra H. Maluleke, Helen E. Struthers, James A. McIntyre, Remco P. H. Peters

**Affiliations:** 1 Anova Health Institute, Johannesburg, South Africa; 2 Anova Health Institute, Tzaneen, South Africa; 3 Department of Viroscience, Erasmus Medical Center, Rotterdam, The Netherlands; 4 Anova Health Institute, Cape Winelands, South Africa; 5 Division of Infectious Diseases & HIV Medicine, Department of Medicine, University of Cape Town, Cape Town, South Africa; 6 School of Public Health and Family Medicine, University of Cape Town, Cape Town, South Africa; Kilimanjaro Centre for Community Ophthalmology, SOUTH AFRICA

## Abstract

Visual impairment is a significant public health concern, particularly in low- and middle-income countries where eye care is predominantly provided at the primary healthcare (PHC) level, known as primary eye care. This study aimed to perform an evaluation of primary eye care services in three districts of South Africa and to assess whether an ophthalmic health system strengthening (HSS) package could improve these services. Baseline surveys were conducted in Cape Winelands District, Johannesburg Health District and Mopani District at 14, 25 and 36 PHC facilities, respectively. Thereafter, the HSS package, comprising group training, individual mentoring, stakeholder engagement and resource provision, was implemented in 20 intervention sites in Mopani District, with the remaining 16 Mopani facilities serving as control sites. At baseline, less than half the facilities in Johannesburg and Mopani had dedicated eye care personnel or sufficient space to measure visual acuity. Although visual acuity charts were available in most facilities, <50% assessed patients at the correct distance. Median score for availability of nine essential drugs was <70%. Referral criteria knowledge was highest in Cape Winelands and Johannesburg, with poor clinical knowledge across all districts. Several HSS interventions produced successful outcomes: compared to control sites there was a significant increase in the proportion of intervention sites with eye care personnel and resources such as visual acuity charts (p = 0.02 and <0.01, respectively). However, engaging with district pharmacists did not improve availability of essential drugs (p = 0.47). Referral criteria knowledge improved significantly in intervention sites (p<0.01) but there was no improvement in clinical knowledge (p = 0.76). Primary eye care in South Africa faces multiple challenges with regard to organisation of care, resource availability and clinical competence. The HSS package successfully improved some aspects of this care, but further development is warranted together with debate regarding the positioning of eye services at PHC level.

## Introduction

Visual impairment (VI) is a significant public health concern, with blindness and moderate to severe VI affecting an estimated 253 million people world-wide [1, 2]. There is a disproportionate burden of VI and blindness in low- and middle-income countries (LMIC) compared to high-income regions [3–5], with socio-economic factors, poor health systems and concomitant human immunodeficiency virus (HIV) and tuberculosis epidemics contributing to the burden in these countries [5–10]. Up to 80% of the burden of VI is preventable and treatable, most often caused by uncorrected refractive error and cataract [2, 11], and 76% of all cases of moderate and severe VI in southern sub-Saharan Africa in 2015 were attributed to these conditions [2]. The World Health Organization’s 2014–2019 global action plan for universal eye health aims to reduce avoidable vision loss [12], thereby curbing the quality-of-life limitations and economic demands associated with visual disabilities [13–16].

In South Africa, eye care is largely provided at the primary healthcare (PHC) level, known as primary eye care [17], with referral to higher level institutions where the need arises. The country does not have a dedicated directorate for eye healthcare, nor is there an integrated eye health promotional policy [18], resulting in inadequate eye care services as has also been described in other African countries [19]. Challenges in the South African eye care programme include insufficient human resources [20, 21], unaffordable or unavailable medication [22, 23], unsatisfactory programme evaluation [18] and inadequate service coverage for Vitamin A supplementation, vision assessments, spectacle provision, cataract surgery and screening for eye complications in patients with diabetes [20–22, 24–28]. In addition, coordination between the different levels of the eye health system is lacking, with poor communication, a complex referral system and problems transporting patients to specialised services [23].

There is global focus on health systems strengthening (HSS) as a key strategy to develop services and ultimately improve health outcomes [29]. With regard to HIV care, for example, integrating HIV services into existing PHC structures, strengthening laboratories and referral linkages, re-training health workers and improving district-level management has been shown to improve HIV care and strengthen wider PHC systems, including improving infrastructure, supervision and patient flow between services [30]. The 2014–2019 global action plan for universal eye health is similarly based on an HSS approach, encompassing the integration of eye care into all levels of the healthcare system, including PHC [12]. However, the value of HSS in the context of primary eye care is unclear and there have been calls for further evaluations to fill the gaps in eye care research [31, 32]. This study aims to address this gap by evaluating a comprehensive HSS strategy to determine if this approach can successfully address shortcomings in primary eye care programmes. Specifically, the study aimed to (a) perform a cross-sectional baseline evaluation of eye services at PHCs in three districts of South Africa with distinct population and healthcare characteristics to determine the overall state of primary eye care in the country and (b) conduct a prospective evaluation of an ophthalmic HSS package to determine if strengthening services at PHC level results in improved organisation of care, resource availability and clinical practice.

## Methods

### Study setting

The baseline evaluation was performed in three districts of South Africa, namely Cape Winelands District in Western Cape Province, Johannesburg Health District in Gauteng Province and Mopani District in Limpopo Province. Cape Winelands falls into the highest Socio-Economic Quintile (SEQ 5) and is among the wealthiest districts in the country [33]. It is sparsely populated, with a density of only 38.5 people/km^2^ [33]. Johannesburg is also in SEQ 5 but is one of the most populous areas of the country (population density of 2 896 people/km^2^) [33]. Mopani is a socio-economically deprived district (SEQ 2) with a relatively low population density of 55.9 people/km^2^ [33]. In terms of PHC management, Cape Winelands performs best, with a PHC supervisor visit rate of 97.2%, while both Johannesburg and Mopani require improvement strategies in this area [33]. The ophthalmic HSS package was only evaluated in Mopani District, as there was an existing HIV and eye disease project in the district which provided the resources and framework in which to implement the intervention.

### Baseline evaluation

A cross-sectional survey of primary eye care services was conducted at a random sample of PHC facilities in each district. Simple random sampling of facilities was performed under epsem, namely using an equal probability of selection method, to sample approximately one-third of facilities in Anova Health Institute-supported sub-districts of Cape Winelands, Johannesburg and Mopani, yielding a sample of 14, 25 and 36 facilities, respectively. The baseline evaluation comprised two components. The first was a self-reported assessment of facility services that was administered to the Facility Manager by a trained interviewer. The assessment included questions regarding organisation of ophthalmic care, availability of essential resources such as a vision acuity (VA) chart, pen torch and direct ophthalmoscope, availability of ophthalmic drugs and data management. The second component was a self‐administered questionnaire for professional nurses who were selected by convenience sampling. The aim was for three nurses at each site to complete the questionnaire, but at some of the smaller facilities (ten (71%) in Cape Winelands and six (17%) in Mopani) it was only possible to recruit two nurses to take part in the survey. Self-reported components of the questionnaire evaluated ophthalmic patient workload, attitude of the nurses toward eye care and knowledge of ophthalmic procedures, such as performing VA, assessing pupillary light reflex, using an ophthalmoscope and administering ophthalmic drugs. The questionnaire also included clinical scenarios to objectively assess knowledge of referral criteria and competence in diagnosing and managing eye conditions, including those related to HIV (S1 Appendix). Both the assessment and questionnaire were based on the PHC Standard Treatment Guidelines and Essential Medicines List, which summarises the eye care services that should be available at PHC level in South Africa according to the National Department of Health [17].

### Intervention: Ophthalmic HSS package

**Fig 1:**
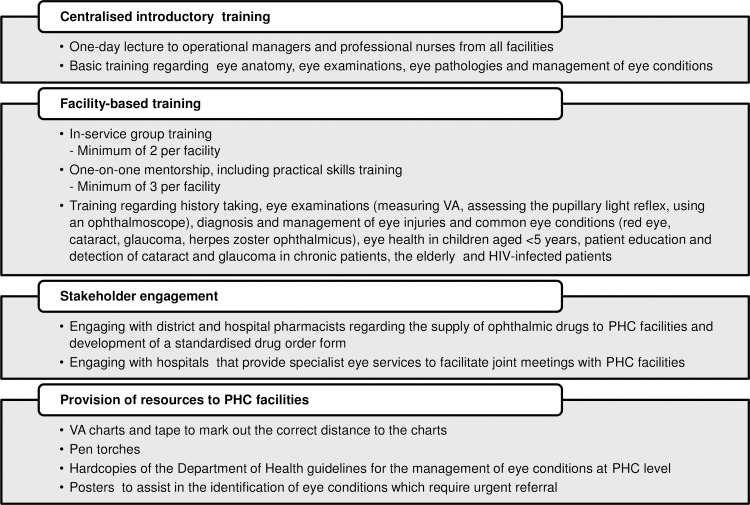
Components of the ophthalmic health systems strengthening intervention. (PHC, Primary Health Care; VA, Visual Acuity).

The prospective study in Mopani District was performed at the same 36 PHC facilities that were sampled in the baseline survey. The ophthalmic HSS package was implemented in two Mopani sub-districts that were selected for operational reasons (Greater Letaba and Greater Tzaneen), incorporating 20 facilities referred to as intervention sites, while the 16 facilities in the remaining three Mopani sub-districts (Greater Giyani, Ba‐Phalaborwa and Maruleng) served as control sites. The package was implemented over a six-month period (January to June 2015) and comprised four components encompassing centralised training, facility-based training, stakeholder engagement and provision of resources (Fig 1).

Firstly, after obtaining approval from senior management in Mopani District, an introductory one-day training was conducted at a centralised location for 110 participants, comprising operational managers and professional nurses from all the intervention sites. The training was delivered in a lecture format by an ophthalmology nurse mentor and focussed on basic clinical knowledge, including anatomy of the eye, eye examinations, eye pathologies and management of eye conditions.

Secondly, facility-based training was provided in the form of in-service group training and one-on-one mentorship. In-service training was conducted by one of two ophthalmology nurse mentors. The aim was to conduct a minimum of two in-service training sessions per facility, which was successful in 17 (85%) facilities; the remaining three sites only allowed a single session to be delivered. The facilities requested that all staff, including professional and enrolled nurses, operational managers and support staff, attend the in-service training to raise awareness of eye care and eye disease. Mentorship visits for professional nurses, involving one-on-one supervision and practical training, were conducted by a single ophthalmology nurse mentor. The aim was to deliver a minimum of three mentorship visits per facility, which was successful in 19 of 20 (95%) intervention sites. Only two visits were performed at the remaining site, as the nurse who was participating in the mentorship programme went on maternity leave and a suitable replacement could not be found. Both in-service training and mentorship visits provided training regarding eye examination, diagnosis of eye conditions (red eye, cataract, glaucoma and herpes zoster ophthalmicus), management of eye conditions and injuries at PHC level, including how to administer ophthalmic drugs and criteria for referral of acute and chronic patients to specialist services, eye health in children and detection of cataract and glaucoma in chronic patients, the elderly and HIV-infected patients.

Thirdly, district and hospital pharmacists were engaged through Drug and Therapeutic Committee meetings regarding ensuring the availability of a standardised list of ophthalmic drugs at PHC level based on the PHC Essential Medicines List [17], namely: topical anaesthetics, sodium chloride, oral pain killers, topical anti-allergic drops, oral anti-allergic medication, topical antibiotics, oral or intra-muscular antibiotics, oral anti-glaucoma drugs and topical miotics. A standardised order form was developed for the PHC facilities that the pharmacists agreed to use in order to prevent stock-outs of ophthalmic drugs. In addition, hospitals that provide specialist eye services were approached by the nurse mentors so that joint meetings with the PHC facilities could be organised in an attempt to improve referral pathways and communication between hospital and PHC staff.

Lastly, basic resources were provided to the intervention sites, as VA charts and pen torches routinely provided to PHC facilities were often misplaced. VA charts and tape to mark out the correct distance to the charts were provided to facilities during the mentoring visits. The ophthalmology nurse mentor put up the VA charts to ensure that there was sufficient space to perform VA and marked the correct patient-chart distance. Pen torches were distributed during in-service training. Hardcopies of the National Department of Health guidelines for the management of eye conditions at PHC facilities were also distributed [34], together with posters that were designed to assist in the identification of eye conditions that require urgent referral.

### Post-intervention evaluations

Effectiveness of the one-day training was evaluated with a pre- and post-training test, undertaken immediately before and after the training. The test included multiple-choice and short answer questions. The impact of the overall intervention package was determined using the same assessment and questionnaire administered at baseline. Within one to three months of completion of all the interventions, the assessments were once again administered to the Facility Managers and the questionnaires were self-administered by three professional nurses per facility. Due to the high turnover of staff at the facilities and the convenience sampling approach, only 80% of nurses who completed the post-intervention questionnaires had also completed the baseline surveys.

### Ethical approval

The study was approved by the Human Research Ethics Committee (Medical) of the University of the Witwatersrand, Johannesburg, South Africa (reference number: M130710) and by the Western Cape, Gauteng and Limpopo Provincial Health Research Committees of the Department of Health. Written informed consent was obtained from all healthcare workers who participated in this study.

### Data analysis

Double data entry was performed using Epi Info^TM^ 3.5.4 (Centers for Disease Control and Prevention, Georgia, USA) and data were then imported into Microsoft Excel and Stata 13.1 (StataCorp LP, Texas, USA) for analysis. Results of the baseline assessments and questionnaires for Cape Winelands, Johannesburg and Mopani were compared using Kruskal-Wallis analysis of variance and χ^2^ or Fisher’s exact tests for continuous and categorical variables, respectively. For the one-day training, paired pre- and post-test scores were compared using a Wilcoxon signed-rank test. Differences in scores were analysed for both the overall score and for test sub-sections (eye anatomy, eye examinations, eye pathologies and management of eye conditions). To assess the impact of the whole intervention package, the change between baseline and post-intervention surveys in intervention sites was compared to the change in control sites using Mann-Whitney and Fisher’s exact tests for continuous and categorical variables, respectively. This analysis was performed at facility-level, the level at which baseline and post-intervention surveys were paired. P < 0.05 was considered significant.

## Results

### Baseline evaluation of primary eye care in South Africa

Baseline assessments demonstrated that organisation of primary eye care does not meet expected standards as specified in the National Department of Health PHC guidelines [17] (Table 1). In terms of staff and infrastructure, less than half the surveyed facilities across all districts had a staff member responsible for ophthalmic care and less than 10% had a designated ophthalmic consulting room. The majority (71%) of Cape Winelands facilities had guidelines for eye screening, compared to less than a third in Johannesburg and Mopani (p < 0.01). Despite the critical need for specialist referral to hospitals, less than 10% of PHC facilities across all districts had meetings with hospitals about eye care and in Johannesburg and Mopani, transport for referral of chronic ophthalmic patients was available in less than half the surveyed facilities. Transport for acute patients was more readily available in all districts, with 100% of Cape Winelands facilities reporting availability of acute patient transport (p < 0.01). Although a significantly higher proportion of referred patients in Cape Winelands returned from hospital with written feedback (p < 0.01), this equated to a median of only 2.5% of referred patients. Eye team visits were only available in Mopani (p < 0.01).

**Table 1:** Baseline evaluation of primary eye care services in three district of South Africa.

	Cape Winelands	Johannesburg	Mopani	p^a^
**Organisation of Care**				
n^b^	14	25	36	
Staff member responsible for ophthalmic care	46.2%	28.0%	13.9%	0.06
Designated ophthalmic consulting room	7.1%	4.0%	0.0%	0.27
Sufficient space to perform VA	76.9%	44.0%	38.9%	0.06
Ability to create a dark environment	69.2%	52.0%	63.9%	0.51
Have guidelines for eye screening	71.4%	16.0%	25.0%	**<0.01**
Meetings with hospitals about eye care	0.0%	0.0%	8.3%	0.29
Transport of referred patients with acute ophthalmic conditions	100%	76.0%	52.8%	**<0.01**
Transport of referred patients with chronic ophthalmic conditions	71.4%	28.0%	44.4%	**0.03**
Returns from hospital with written feedback, median (range)	2.5% (0.0–100)	0.0% (0.0–2.0)	0.0% (0.0–20.0)	**<0.01**
Eye teams visit the facility	0.0%	0.0%	75.0%	**<0.01**
**Essential Resources & Drugs**				
n^b^	14	25	36	
Distance VA chart available	100%	68.0%	80.6%	0.06
Correct distance from patient to VA chart	46.2%	0.0%	24.1%	**<0.01**
Pen torch available	38.5%	72.0%	8.3%	**<0.01**
Direct ophthalmoscope available	78.6%	16.0%	55.6%	**<0.01**
Essential drugs available, median (range)^c^	66.7% (44.4–100)	66.7% (44.4–66.7)	55.6% (22.2–77.8)	**<0.01**
Stock-outs of essential drugs in previous 3 months	15.4%	44.0%	50.0%	0.09
**Clinical Practice**				
n^b^	32	75	102	
Saw an ophthalmic patient <6 months ago	90.3%	93.3%	98.0%	0.10
Estimated ophthalmic patients seen per month, median (range)	5.0 (0.0–13.0)	15.0 (1.0–50.0)	14.5 (2.0–99.0)	**<0.01**
Enjoy serving ophthalmic patients^d^	16.1%	38.7%	49.0%	**<0.01**
Comfortable managing ophthalmic patients^d^	9.7%	29.3%	34.3%	**0.03**
Consider ophthalmic knowledge adequate^d^	6.3%	21.3%	5.9%	**<0.01**
Know how to perform VA^d^	75.0%	53.3%	35.3%	**<0.01**
Know how to assess the pupillary light reflex^d^	62.5%	38.7%	11.8%	**<0.01**
Know how to use an opthalmoscope^d^	45.2%	30.7%	10.8%	**<0.01**
Know how to administer topical eye drugs^d^	71.9%	49.3%	36.3%	**<0.01**
Aware of referral criteria for ophthalmic patients	83.3%	93.3%	58.8%	**<0.01**
Knowledge of referral criteria: objectively evaluated^e^	84.4%	96.0%	55.9%	**<0.01**
Clinical knowledge: objectively evaluated^f^	15.6%	25.3%	12.7%	0.09

All variables are self-reported unless otherwise indicated. VA, visual acuity.

^a^ Statistically significant differences are shown in bold.

^b^ Assessments evaluating Organisation of Care and Essential Resources & Drugs were administered at facility level (n = facilities); Questionnaires evaluating Clinical Practice were administered at an individual level (n = individuals).

^c^ Percentage of the following nine drugs that were available in each facility: topical anaesthetics, sodium chloride, oral pain killers, topical anti-allergic drops, oral anti-allergic medication, topical antibiotics, oral or intra-muscular antibiotics, oral anti-glaucoma drugs and topical miotics.

^d^ Self-reported using a scale of 1 to 5, where 1 = not at all, 3 = fine and 5 = very much. Percentage represents the number of ratings of 4 or 5 out of the total number of ratings.

^e^ Percentage who answered 2 or more questions regarding referral criteria correctly out of 3.

^f^ Percentage who answered 6 or more clinical questions correctly out of 11, namely scored >50%.

Although distance VA charts were available in the majority of facilities across all districts, less than half the facilities reported that patients were assessed at the correct distance, with not a single Johannesburg facility reporting correct use of the VA chart (p < 0.01) (Table 1). Nevertheless, over two thirds of Johannesburg facilities had a pen torch, higher than in the other districts (p < 0.01). The median score for availability of nine essential drugs required for primary eye care was 67% in Cape Winelands and Johannesburg, and significantly lower at 56% in Mopani (p < 0.01). Drug stock-outs were particularly problematic in Johannesburg and Mopani, with 40–50% of facilities reporting stock-outs in the three months prior to the survey. With regard to data management, less than 5% of facilities in any district collected ophthalmic indicators.

Virtually all nurses who completed questionnaires at baseline were female (97%, 95% and 88% in Cape Winelands, Johannesburg and Mopani, respectively) and median age was 49, 46 and 42 years, respectively. The vast majority of respondents had seen an ophthalmic patient in the six months prior to the survey (Table 1). Although a higher proportion of nurses in Mopani reported enjoying and being comfortable with eye patients compared to the other districts (p < 0.01 and = 0.03, respectively), they had the lowest self-reported knowledge across all ophthalmic procedures. Respondents from Cape Winelands consistently reported the highest knowledge, ranging from 40% to 75% depending on the procedure. Objective evaluation of referral criteria knowledge was highest in Cape Winelands and Johannesburg, with 84% and 96% of respondents scoring over 67%, respectively, though far fewer correctly answered all three of the referral questions (19%, 43% and 9% in Cape Winelands, Johannesburg and Mopani, respectively, p < 0.01). Clinical knowledge objectively assessed with the clinical questions was poor, with less than a third of respondents scoring over 50% and no significant difference across the districts (p = 0.09).

### Post-intervention evaluation: Impact of the ophthalmic HSS package

The one-day training proved effective in increasing knowledge scores, with the overall test score increasing from a median of 46% pre-training to 66% post-training (p < 0.01). Similarly, in all test sub-sections there was a significant increase in score in the post-training test (p < 0.01 for eye anatomy, eye examination, eye pathologies and management of eye conditions).

Post-intervention assessments were successfully administered in all 20 intervention sites and 16 control sites within one to three months of completion of the HSS intervention (Table 2). There were no statistically significant differences between the intervention and control sites at baseline, but a number of significant improvements were found in the intervention sites after implementation of the HSS package. With regard to the organisation of eye care, there was a significant increase in the proportion of intervention sites with a staff member responsible for ophthalmic care (p = 0.02), eye screening guidelines (p < 0.01) and transport for acute ophthalmic patients (p < 0.01) compared to control sites. For the latter two indicators, however, the control sites reported 0% availability of these services post-intervention, which was a substantial and unexpected decrease in service provision. Even if it was assumed that this was a reporting bias and there was in fact no change in the control sites, the increase in the intervention sites remained significant (p < 0.01 for both availability of guidelines and acute transport). Intervention sites also reported an increase in meetings with hospitals about eye care (p = 0.04), but this finding was no longer significant if the 0% post-intervention measure in the control sites was assumed inaccurate (p = 0.24 assuming no change in the control sites). This corroborates reports from nurse mentors regarding difficulties in arranging hospital meetings due to tension between PHC-level and hospital-level ophthalmic staff.

**Table 2:** Baseline and post-intervention surveys in Mopani District to assess the impact of a health system strengthening support package to improve primary eye care.

	Intervention Sites		Control Sites		
	Baseline	Post	Baseline	Post	p^a^
**Organisation of Care**					
n^b^	20	20	16	16	
Staff member responsible for ophthalmic care	10.0%	40.0%	18.8%	12.5%	**0.02**
Designated ophthalmic consulting room	0.0%	5.0%	0.0%	0.0%	1.00
Sufficient space to perform VA	40.0%	55.0%	37.5%	43.8%	1.00
Ability to create a dark environment	65.0%	73.7%	62.5%	81.3%	0.48
Have guidelines for eye screening	20.0%	75.0%	31.3%	0.0%	**<0.01**
Meetings with hospitals about eye care	0.0%	15.0%	18.8%	0.0%	**0.04**
Transport of referred patients with acute ophthalmic conditions	50.0%	70.0%	56.3%	0.0%	**<0.01**
Transport of referred patients with chronic ophthalmic conditions	45.0%	40.0%	43.8%	0.0%	0.17
Returns from hospital with written feedback, median (range)	0.0% (0.0–1.0)	1.0% (0.0–100)	0.0% (0.0–20.0)	5.0% (0.0–20.0)	0.74
Eye teams visit the facility	75.0%	75.0%	75.0%	62.5%	0.88
**Essential Resources & Drugs**					
n^b^	20	20	16	16	
Distance VA chart available	75.0%	100%	87.5%	56.3%	**<0.01**
Correct distance from patient to VA chart	26.7%	72.2%	21.4%	33.3%	0.19
Pen torch available	0.0%	70.0%	18.8%	0.0%	**<0.01**
Direct ophthalmoscope available	50.0%	47.4%	62.5%	12.5%	0.10
Essential drugs available, median (range)^c^	55.6% (22.2–66.7)	55.6% (22.2–77.8)	55.6% (22.2–77.8)	55.6% (33.3–88.9)	0.47
Stock-outs of essential drugs in previous 3 months	55.0%	33.3%	43.8%	53.3%	0.21
**Clinical Practice**					
n^b^	57	60	45	48	
Saw an ophthalmic patient <6 months ago	100%	93.1%	95.6%	97.9%	0.08
Estimated ophthalmic patients seen per month, median (range)	12.0 (3.0–50.0)	10.0 (0.0–80.0)	15.0 (2.0–99.0)	15.0 (2.0–80.0)	0.16
Enjoy serving ophthalmic patients^d^	49.1%	50.8%	48.9%	25.5%	0.08
Comfortable managing ophthalmic patients^d^	36.8%	43.1%	31.1%	25.0%	0.28
Consider ophthalmic knowledge adequate^d^	7.0%	18.6%	4.4%	12.5%	0.53
Know how to perform VA^d^	33.3%	63.3%	37.8%	14.6%	**<0.01**
Know how to assess the pupillary light reflex^d^	7.0%	36.7%	17.8%	14.6%	**<0.01**
Know how to use an ophthalmoscope^d^	5.3%	18.3%	17.8%	8.3%	**0.02**
Know how to administer topical eye drugs^d^	33.3%	57.9%	40.0%	37.5%	**0.03**
Aware of referral criteria for ophthalmic patients	59.6%	88.1%	57.8%	72.9%	0.33
Knowledge of referral criteria: objectively evaluated^e^	56.1%	80.0%	55.6%	33.3%	**<0.01**
Clinical knowledge: objectively evaluated^f^	14.0%	16.7%	11.1%	8.3%	0.76

All variables are self-reported unless otherwise indicated. VA, visual acuity.

^a^ p for change in intervention sites versus change in control sites. Statistically significant differences are shown in bold.

^b^ Assessments evaluating Organisation of Care and Essential Resources & Drugs were administered at facility level (n = facilities); Questionnaires evaluating Clinical Practice were administered at an individual level (n = individuals) and then aggregated to facility level for analysis.

^c^ Percentage of the following nine drugs that were available in each facility: topical anaesthetics, sodium chloride, oral pain killers, topical anti-allergic drops, oral anti-allergic medication, topical antibiotics, oral or intra-muscular antibiotics, oral anti-glaucoma drugs and topical miotics.

^d^ Self-reported using a scale of 1 to 5, where 1 = not at all, 3 = fine and 5 = very much. Percentage represents the number of ratings of 4 or 5 out of the total number of ratings.

^e^ Percentage who answered 2 or more questions regarding referral criteria correctly out of 3.

^f^ Percentage who answered 6 or more clinical questions correctly out of 11, namely scored >50%.

Interventions to improve the organisation of other components of eye care services proved to be unsuccessful (Table 2)–there was no significant improvement in the proportion of intervention sites with sufficient space to perform VA even though this was targeted as part of the intervention (p = 1.00), nor was there improvement in the proportion of referred patients returning from hospital with written feedback (p = 0.74). The support package did not include infrastructure or district-level resource improvements, and there were therefore, as expected, no changes in the availability of a designated ophthalmic consulting room, the ability to create a dark environment, additional avenues for transport of chronic patients or increased access to eye teams (p = 1.00, 0.48, 0.17 and 0.88, respectively).

Providing distance VA charts and pen torches to the intervention sites proved to be a successful intervention for increasing the availability of these resources (p < 0.01 for both) (Table 2). However, marking the correct distance between the patient and the VA chart in the intervention sites did not result in a significant improvement in this indicator versus control facilities (p = 0.19). Furthermore, engaging with district and hospital pharmacists did not improve availability of essential drugs or significantly prevent stock-outs (p = 0.47 and 0.21, respectively).

Over 90% of nurses responding to the post-intervention questionnaire saw an ophthalmic patient in the six months prior to the survey, equivalent to the findings at baseline in both the intervention and control sites (p = 0.08) (Table 2). Despite the training and mentoring in intervention sites, there was no significant improvement in perceptions of nurses toward eye care in terms of enjoying or being comfortable with ophthalmic patients (p = 0.08 and 0.28, respectively). Although nurses in the intervention sites did not consider their ophthalmic knowledge significantly improved (p = 0.53), self-reported knowledge regarding specific procedures, namely performing VA, assessing the pupillary light reflex, using an ophthalmoscope and administering topical eye drugs significantly increased compared to control sites (p < 0.01, < 0.01, 0.02 and 0.03 respectively). Awareness of referral criteria increased to the same degree in intervention and control facilities (p = 0.33), but objective assessment of referral criteria knowledge indicated a significant improvement in the intervention sites (p < 0.01). Conversely, nurses in the intervention facilities showed no improvement in clinical knowledge compared to those in the control sites (p = 0.76).

## Discussion

This study demonstrates poor levels of primary eye care services in South Africa, with challenges in organisation of care, availability of essential resources and clinical knowledge of PHC workers. Of the three districts evaluated, Cape Winelands was better organised and resourced and had higher self-reported knowledge ratings for ophthalmic procedures, likely a reflection of it being a wealthier district with a relatively small population, coupled with excellent PHC supervision [33]. A comparable survey of PHC workers in a rural area of Free State Province, South Africa published in 2000 found notably similar results to this study, particularly with regard to findings in Johannesburg and Mopani [23]. The survey noted problems with transportation of patients to specialist services, poor feedback from these services, lack of resources, problems with availability of medication and inadequate PHC-worker knowledge [23], highlighting the lack of substantial improvements in primary eye care in many districts of South Africa in the last 15 to 20 years. Other than this study there have been few, if any, comprehensive assessments of primary eye care services in South Africa. Even routine monitoring and evaluation of eye services has been lacking, with some eye care managers reporting no monitoring and evaluation methods [18] and one study noting a complete absence of eye health data collection at PHC level [35] in agreement with findings in this study. The paucity of eye care data in South Africa is a critical problem which hinders effective monitoring and ultimately improvement of eye health services in the country. Challenges regarding lack of data have also been described in other African settings [36], further emphasising the need to establish effective systems to monitor and evaluate eye care services in accordance with the World Health Organisation’s global action plan for universal eye health [12].

It is evident that South Africa’s primary eye care services lack the organisation and resources to address the leading causes of VI, namely, uncorrected refractive error and cataract [2, 11]. Identification and correction of refractive error requires vision screening as a first step. We found that distance VA charts were not available in all facilities and where they were available, patients were most often assessed at the incorrect distance, compromising the VA measurements. It is not surprising that vision screening is inadequate, as 81% (43/53) of South African provincial health directorate managers reported that they do not include vision screening in their health promotional programs [18], minimising the importance of this service. Similar problems with VA screening are evident in other African countries, where healthcare workers have been shown to lack competence in assessing VA, including use of the incorrect patient-chart distance [37, 38]. Even if VA screening were to be correctly performed, glasses are not routinely provided at PHC facilities in South Africa which would limit access to any correction of refractive errors. With regard to treatment of cataracts, PHC facilities should refer patients to higher-level institutions for vision-restoring surgery. However, surgery capacity in South Africa is markedly inadequate and there is a lack of commitment by senior management to increase cataract surgery rates [21]. Even when cataract surgery is available, there are barriers to uptake of surgery at PHC level, with this study noting inadequate transport for referred patients, particularly for those with chronic conditions such as cataracts, and virtually no meetings between PHC facilities and hospitals to facilitate an integrated and coordinated referral system. In addition, there is a lack of awareness regarding the possibility of treatment [6, 39, 40], suggesting that the role of primary eye care in acting as a gateway to specialist services is lacking.

Interestingly, we found that self-reported knowledge, clinical knowledge score and comfort with treating ophthalmic patients did not necessarily correspond–respondents from Cape Winelands had the highest self-reported knowledge for specific ophthalmic procedures, but had low clinical knowledge scores and were least comfortable managing ophthalmic patients compared to respondents in the other two districts. In contrast, respondents from Mopani who had the lowest levels of knowledge, both by self-report and knowledge score, were most comfortable with ophthalmic care. Although desirability bias cannot be ruled out, this may suggest that lack of knowledge is indicative of a lack of awareness regarding the complexity of eye care, which may therefore promote greater comfort in providing this service. This may contribute to the practice of PHC workers in providing ophthalmic services beyond their capacity, even if knowledge, skills and training are lacking [37, 38]. Providing clinical care without the necessary knowledge is of no benefit to patients and will ultimately compromise patient confidence in primary eye care services [37].

**Fig 2:**
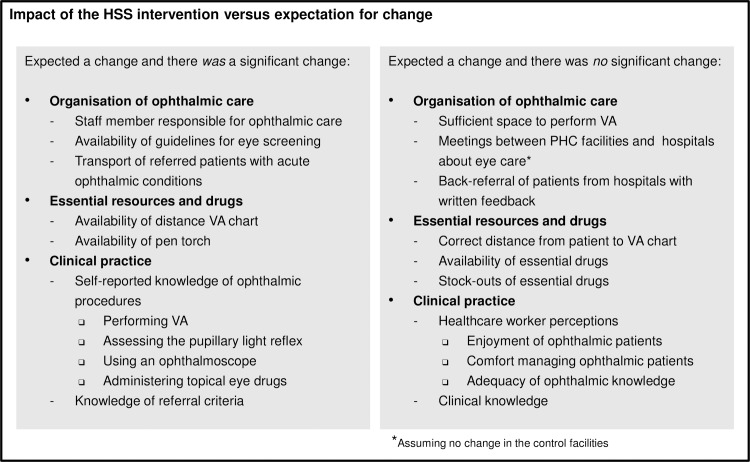
Impact of the HSS intervention in Mopani District versus expectation for change. (HSS, Health Systems Strengthening; PHC, Primary Health Care; VA, Visual Acuity).

Based on our experience in HIV healthcare support, we piloted and successfully implemented an ophthalmic HSS package designed to strengthen primary eye care services by improving organisation of care, availability of essential resources and clinical practice. Day-training significantly improved knowledge scores immediately post-training, in agreement with previous findings that training focussed on primary eye care does improve knowledge scores in the short-term [41]. The overall intervention package, however, produced mixed results, with some interventions producing significant improvements and others having no significant impact, even though there was an expectation for change (Fig 2). With regard to organisation of care, successful outcomes included availability of guidelines for eye screening and transport for acute patients, the latter most likely due to raised awareness of the importance of early referral of ophthalmic emergencies and access to already available emergency transport services at PHC facilities. As expected, distribution of resources such as VA charts and pen-torches improved their availability post-intervention, in agreement with findings from a study of enhanced supervision with a similarly short follow-up period of six months [42], though success in the longer term is questionable, with very low levels of these supplies being reported in other African settings after a follow-up period of two years [43]. Surprisingly, the proportion of intervention facilities with sufficient space to perform VA and the correct patient-chart distance did not improve significantly compared to the control sites. Although there was a trend to improved outcomes in these indicators after the intervention, it is likely that charts were affixed in common areas such as passage-ways due to the space limitations in many clinics, which may have been perceived as inappropriate for performing VA and where tape marking patient distance may have been removed by someone who was unaware of its purpose. In addition, efforts to engage with stakeholders of eye care services including hospitals and pharmacists proved unsuccessful–difficulties arose in arranging meetings with hospitals due to tension between the different cadres of staff working at the hospitals and PHC facilities, and pharmacists were unable to deliver drugs to PHC facilities due to provincial stock-outs of essential drugs during the study period. The success of the HSS intervention in improving organisation of eye care and availability of essential resources was thus limited by health-system barriers including poor clinic infrastructure, ineffective communication between services and problematic supply-chain management.

Similar to findings regarding organisation of care and essential resources, the HSS intervention produced mixed results with regard to clinical practice (Fig 2). Although self-reported knowledge and knowledge of referral criteria increased significantly in intervention sites, there was no significant improvement in how comfortable nurses felt in managing ophthalmic patients or in their clinical knowledge scores. An evaluation of PHC workers in Tanzania two to three years after a four-day training programme similarly found that training was more effective in raising awareness of eye health than in conveying clinical skills, with participants better able to appropriately refer a patient than to correctly diagnose a condition [41]. Similarly, other studies have demonstrated lack of clinical competence in healthcare workers despite eye care training [37, 38] and have questioned whether the modest improvements in knowledge and skills following enhanced supervision are of any clinical significance [43]. Even after a short follow-up period of six months, a study in Tanzania found that enhanced supervision only improved specific skills to modest levels, with no significant improvement in the overall skills score [42]. The potential for training to improve clinical skills of PHC workers may be limited due to the low numbers of ophthalmic patients who present at PHC compared to patients with other conditions, making it difficult to gain sufficient experience for the retention of knowledge and skills [37]. The findings of this study add to the body of evidence which questions whether primary eye care can indeed provide an acceptable quality of service or meet the needs of target populations [44, 45]. As previously suggested, primary eye care might therefore be better suited to eye health education and referral of patients using well-defined guidelines as opposed to providing diagnostic and clinical management services [43, 46].

The ophthalmic HSS package implemented in this study warrants further development to improve its success, as the burden of eye disease, majority of which is age-related, is expected to rise in the coming years, consistent with a growing population with increasing life expectancy [47]. In the context of the HIV epidemic in South Africa and other LMICs, effective treatment with antiretroviral therapy will further contribute to the ageing population and will increase the occurrence of ocular disease, particularly among individuals receiving long-term treatment [8, 48]. While the focus of the PHC ophthalmic HSS package may need to shift to eye health education and referral pathways, additional improvements are required, some of which may be derived from successful HSS interventions in the HIV programme. An intervention in Mozambique highlighted the importance of understanding the structure of the existing health system in terms of the geographic units of administration and levels of care, as a successful HSS package needs to be implemented at the key organisational division in the healthcare system [30]. In addition, strategies to improve district-level management and to strengthen support services through provision of resources and training, neither of which was targeted in the ophthalmic HSS package, should be considered [30].

There are several limitations to this study. Firstly, post-intervention evaluations were performed within one to three months after completion of the interventions which were rolled out over a six-month period, and it is possible that some health system changes would only have been evident after a longer time. Secondly, turnover of healthcare workers in PHC clinics is unavoidable, with nurses resigning, being transferred to night duty or taking sick- or maternity-leave. It was therefore not always possible to provide mentoring to the same nurse at consecutive visits or to ensure that the same nurses completed the baseline and post-intervention questionnaires. In these cases, post-intervention changes were reflective of in-service training performed for all staff and knowledge-sharing between nurses who were mentored and their colleagues, which should occur routinely in PHC facilities. Thirdly, the post-intervention assessments in the control sites produced some unexpected results, with several indicators dropping to 0%. Operational factors were investigated, but there was no obvious explanation as to why such substantial decreases in service provision were reported. This raises questions regarding the accuracy of the data and the contribution of reporting biases. Fourth, the HSS intervention package was only implemented in Mopani, the poorest district evaluated in this survey. Impact of the intervention may have been different in the other two districts, particularly in Cape Winelands which had better baseline findings. Fifth, most of the findings were self-reported which may have led to recall bias in some variables, for example, the number and frequency of ophthalmic patients seen in the PHC facility in recent months. Finally, it would have been beneficial to evaluate the perceptions of ophthalmic patients regarding primary eye care services.

## Conclusions

Primary eye care in South Africa is faced with multiple challenges in terms of organisation of care, availability of resources and clinical competence, and is not provided to the standard that is required by the National Department of Health. The novel ophthalmic HSS package implemented in this study produced mixed results, with some components of the package proving successful in strengthening basic eye care services and others producing no significant improvements. This approach warrants further development, as is the case with all HSS strategies. Nevertheless, further debate is required regarding the organisation and positioning of eye services at PHC level both in South Africa and in other LMICs.

## Supporting information

S1 Appendix Clinical scenarios used to objectively assess professional nurses’ understanding of referral criteria and clinical knowledge. (PDF)

S2 Appendix Data collected during the baseline and post-intervention evaluations. (XLSX)
