# Supplementary material for: Strengthening primary eye care in South Africa: An assessment of services and prospective evaluation of a health systems support package
Source: PLoS One. 2018 May 14;13(5):e0197432. doi: 10.1371/journal.pone.0197432 (PMC5951550; doi:10.1371/journal.pone.0197432)
Supplement: S1 Appendix — (PDF) [file pone.0197432.s001.pdf]

S1 Appendix. Clinical scenarios used to objectively assess professional nurses' understanding of referral criteria and clinical knowledge.

Referral criteria knowledge

Would you refer a patient with:

1) Painful eye with good visual acuity and a history of eye injury
2) Red eye with good visual acuity
3) Red eye in a new-born

Clinical knowledge

1) What would be the most likely diagnosis in a patient with a unilateral (affecting only one eye) painful red eye and photophobia (intolerance to light)
    a. Conjunctivitis
    b. Keratitis
    c. Glaucoma
    d. Uveitis

2) What would be the most likely diagnosis in a patient with bilateral (affecting both eyes) red eyes and watery discharge only
    a. Allergic conjunctivitis
    b. Bacterial conjunctivitis
    c. Viral conjunctivitis
    d. None of the above

3) What is the most common cause of infectious conjunctivitis (conjunctival infection)
    a. Bacteria
    b. Virus
    c. Fungus

4) What would be the most likely diagnosis in a patient with a unilateral (affecting only one eye) painful red eye, photophobia (intolerance to light), and a history of injury to the eye
    a. Conjunctivitis
    b. Keratitis
    c. Glaucoma
    d. Uveitis

5) Which medicine would you prescribe for a bacterial conjunctivitis (conjunctival infection)
   a. Antibiotic ointment 3 times a day
   b. Antibiotic ointment 6 times a day
   c. Antibiotic drops 3 times a day
   d. Antibiotic drops 3 times a day

6) What would be the most common condition after ART initiation
   a. Conjunctivitis
   b. Keratitis
   c. Uveitis
   d. Glaucoma

7) How would you classify a patient with visual acuity right eye of 3/60 and visual acuity left eye 4/6
   a. Normal vision
   b. Visually impaired
   c. Severe visually impaired
   d. Blind
   e. I don't know what 3/60 and 4/6 means

8) In which cases would you prescribe chloramphenicol: 1) keratitis, 2) allergic conjunctivitis, 3) cataract, 4) foreign body, 5) infectious conjunctivitis
   a. All of the above
   b. 1,2,3
   c. 1,2,4,5
   d. 1,4,5
   e. 2,4,5
   f. None of the above

9) What would you do if the anterior chamber was shallow in a patient with diabetes mellitus and decreased visual acuity
   a. Refer patient
   b. Dilate and perform fundoscopy

10) Ocular diseases in HIV-infected individuals are
   a. Less common
   b. As common as in HIV-uninfected individuals
   c. More common

11) Which condition is associated with or more common in HIV-infection: 1) CMV retinitis, 2) squamous cell carcinoma, 3) conjunctival Kaposi sarcoma, 4) primary glaucoma

a. All of the above
b. 1,2
c. 1,3,4
d. 2,3,4
e. 2,4
f. None of the above
